# Detectable HIV-1 in semen in individuals with very low blood viral loads

**DOI:** 10.1186/s12985-020-01300-6

**Published:** 2020-03-05

**Authors:** Samuel Mundia Kariuki, Philippe Selhorst, Jennifer Norman, Karen Cohen, Kevin Rebe, Carolyn Williamson, Jeffrey R. Dorfman

**Affiliations:** 1 Division of Immunology, Department of Pathology, University of Cape Town, Anzio Rd, Observatory, Cape Town, 7925 South Africa; 2 International Centre for Genetic Engineering and Biotechnology, Cape Town, South Africa; 3 Department of Biological Sciences, School of Science, University of Eldoret, Eldoret, Kenya; 4 Division of Medical Virology, Department of Pathology, University of Cape Town, Cape Town, South Africa; 5 Virology Unit, Department of Biomedical Sciences, Institute of Tropical Medicine, Antwerp, Belgium; 6 Division of Clinical Pharmacology, Department of Medicine, University of Cape Town, Cape Town, South Africa; 7 Anova Health Institute, Cape Town, South Africa; 8 Division of Infectious Diseases and HIV Medicine, Department of Medicine, University of Cape Town, Cape Town, South Africa; 9 Institute for Infectious Disease and Molecular Medicine, University of Cape Town, Cape Town, South Africa; 10 National Health Laboratory Service, Johannesburg, South Africa; 11 Division of Virology, Faculty of Medicine and Health Sciences, Stellenbosch University, Parow, South Africa

**Keywords:** HIV-1, Semen, Blood, Viral load

## Abstract

**Background:**

Several reports indicate that a portion (5–10%) of men living with HIV-1 intermittently shed HIV-1 RNA into seminal plasma while on long term effective antiretroviral therapy (ART). This is highly suggestive of an HIV-1 reservoir in the male genital tract. However, the status of this reservoir in men living with HIV-1 who are not under treatment is underexplored and has implications for understanding the origins and evolution of the reservoir.

**Finding:**

Forty-three HIV-1 positive, antiretroviral therapy naïve study participants attending a men’s health clinic were studied. Semen viral loads and blood viral loads were generally correlated, with semen viral loads generally detected in individuals with blood viral loads > 10,000 cp/ml. However, we found 1 individual with undetectable viral loads (<20cp/ml) and 2 individuals with very low blood viral load (97 and 333cp/ml), but with detectable HIV-1 in semen (485–1157 copies/semen sample). Blood viral loads in the first individual were undetectable when tested three times over the prior 5 years.

**Conclusions:**

Semen HIV-1 viral loads are usually related to blood viral loads, as we confirm. Nonetheless, this was not true in a substantial minority of individuals suggesting unexpectedly high levels of replication in the male genital tract in a few individuals, despite otherwise effective immune control. This may reflect establishment of a local reservoir of HIV-1 populations.

## Introduction

Several reports in a range of settings globally indicate that a portion (5–10%) of men living with HIV-1 intermittently shed HIV-1 RNA into seminal plasma while on long term effective antiretroviral therapy (ART) [1–5]. In a series of observational studies, viral suppression with antiretroviral therapy is associated with no detectable risk of transmission [6], even in populations of men having sex with men with high rates of sexually transmitted infections and evidence for high rates of high risk sex [7]. The reason that HIV-1 RNA shed into semen is not associated with a detectable risk of transmission is unclear. Plausibly, the proportion of virions that are viable in this context is low [8] and thus an infectious dose of viable virions is not reached. Nonetheless, the presence of HIV-1 RNA in semen is highly suggestive of an HIV-1 reservoir in the male genital tract [9]. However, the status of this reservoir in men living with HIV-1 not under treatment is underexplored and has implications for understanding the origins and evolution of the reservoir.

The presence of HIV-1 RNA during effective ART is very likely indicative of persisting HIV-1 production in the male genital tract because the half-life of HIV-1 virions in serum is less than 8 h [10]. The reason production of HIV-1 is able to persist in the male genital tract is not clear. In some cases, penetration of ART drugs into the male genital tract may be incomplete, but the picture is complex and a simple association is not obvious [11].

In this report, we studied men living with HIV-1 at a Men’s clinic in Cape Town, South Africa who were not yet on ART. We found individuals who were apparently capable of suppressing their viral loads in the blood circulation, but nonetheless did shed HIV-1 RNA into their semen.

## Materials and methods

### Study participants

Forty-three HIV-1 positive, ART-naive study participants were recruited between June 2015 and January 2017 from ANOVA Health’s Ivan Toms Health4Men clinics in Woodstock, Green Point or Khayelitsha, all in Cape Town, South Africa. Study participants were scheduled for sample collection and interview when the clinic was otherwise closed and were asked to abstain from sexual activity for 72 h prior to sample collection. For each study participant, both blood and semen samples were collected during a single visit. Study participants were interviewed and clinical records were reviewed to identify current and recent sexually transmitted infections (STIs) and clinical history. Study participants all self-reported to be ART naive and none previously received ART from Ivan Toms Health4Men clinics.

### Sample handling and testing

The entire specimen of semen was diluted 1:1 with phosphate buffered saline (PBS) and then underlaid with 19% Nycodenz (Axis-Shield PoC AS, Oslo, Norway) in PBS with penicillin/streptomycin and centrifuged (1000 g, 20 min) to separate seminal plasma from sperm and other cells. Seminal plasma was recovered, filtered (0.8 μm) and HIV-1 was concentrated by centrifugation (100,000 g, 1 h 4 °C). The pellet was resuspended in 200 μl of PBS, 40 μl of which was diluted to 500 μl and assayed for viral load. The limit of detection under these conditions was 100cp (copies)/ml, corresponding to 250cp/semen sample. Viral loads were measured in a clinically accredited laboratory (National Health Laboratory Service, Groote Schuur Hospital, Cape Town) by the Roche COBAS AmpliPrep/TaqMan methodology. Blood samples were scored as undetectable only if the viral load was undetectable at the time of collection and also in a confirmatory test performed later on frozen blood plasma.

## Results and discussion

**Fig. 1:**
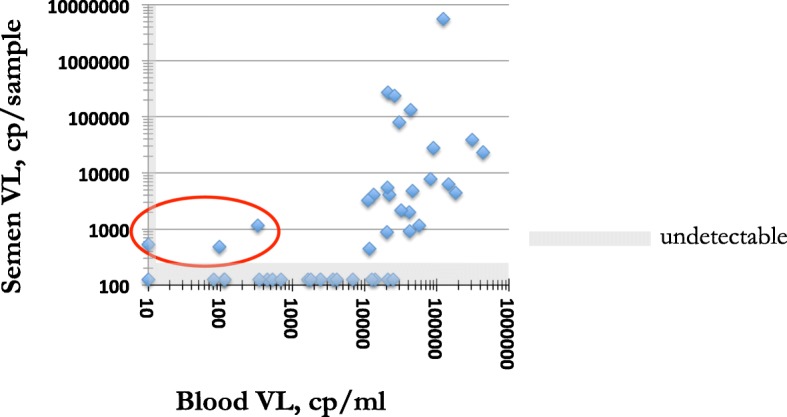
Display of blood viral load (cp/ml) vs semen viral load (cp/sample) for the 43 individual men in this study. Values plotted as 10cp/ml or 125cp/sample reflect undetectable viral loads. The red circle encloses the 3 individuals with detectable viral loads in semen but low or undetectable viral loads in blood

Median age was 29 years (IQR 25–36); median CD4 ^+^ T cell count was 519 cells/μl (IQR 370–631), and median blood viral load was 4.10 log_10_ cps/ml (IQR 2.69–4.56). Generally, HIV-1 was detected in semen in samples with blood viral load > 10,000cp/ml (Fig. 1) and for those samples, the log_10_ semen viral loads correlated moderately with the log_10_ blood viral loads (R^2^ = 0.1556, *p* = 0.026). This is in line with previous studies showing a similar correlation, with thresholds ranging from 2000 to 10,000cp/ml [12–16] (reviewed in [17]). Some studies comparing blood to semen viral loads had very few individuals with blood viral loads below 10,000 cp/ml [18–20], possibly because they included many individual with low CD4^+^ T cell counts.

The rough correlation between the two measures and apparent threshold effect suggests that HIV-1 in the male genital tract is ultimately fed from the larger HIV-1 populations in blood/general circulation (Kariuki SM, P. S, Anthony A, Matten D, Abrahams MR, Martin DP, Ariën KA, Rebe K, Williamson C, Dorfman JR: Compartmentalization and clonal amplification of HIV-1 in the male genital tract characterized using next generation sequencing, submitted for publication). This is in contrast to what is found by Klein et al [21] in the genital tracts of women with early stage HIV-1 infections. There, the blood populations appeared to be derived from those found in the genital mucosa. It is possible that HIV-1 could reach the male genital tract from the blood circulation in cases in which external male sex organs were the original site of inoculation. Additionally, there may be differences in the site of the original HIV-1 inoculation in the participants in the present study, perhaps due to high intravenous drug use in the population from which our study participants were drawn [22] and/or inoculation via the rectum. In such cases, HIV-1 reaching the male genital tract presumably did so via the blood circulation.

More interestingly, we found 1 individual with undetectable viral loads (<20cp/ml) and 2 individuals with very low blood viral load (97 and 333cp/ml), but with detectable HIV-1 in semen, ranging from 485 to 1157 copies/semen sample (Fig. 1). These did not fit the pattern of correlation between blood and semen viral loads. The individual that had undetectable blood viral load, had undetectable blood viral loads when tested previously, approximately 3 and 5 years prior. CD4^+^ T cell counts of these three individuals (range 403–1123 cells/μl) were not significantly different from the other study participants (*p* = 0.14), nor was age (*p* = 0.51). None of the three individuals reported sexually transmitted infections (STIs) and no STIs were detected upon clinical folder inspection or clinician interviews on the day of sample collection. The small sample size prevents meaningful statistical comparison.

The detection of HIV-1 viral loads in seminal plasma in these individuals suggests that even highly effective immune responses maintained for extended periods are not always sufficient to suppress shedding of HIV-1 RNA into seminal plasma. A review of prior data reveal that other studies may have observed one or two individuals each with such patterns [12–16] (Figure S1). None of these studies commented upon these individuals. A recent report [23] studied 10 HIV-1 controllers and measured secretion of HIV-1 RNA into seminal plasma. HIV-1 RNA over 40 cp/ml in semen of the HIV-1 controllers was detected, but only in individuals with relatively high contemporaneous or 30-day prior blood viral loads (685–5750 cp/ml). It thus seemed possible from that study that the HIV-1 found in semen could have been recently sourced from the systemic circulation. Here, we describe individuals with lower contemporaneous blood viral loads, including one with three blood viral loads below the detection limit over 5 years.

Individuals with an HIV-1 reservoir in the male genital tract may be more common than we observe because (i) they would be less obvious when reservoir-derived HIV-1 RNA is intermingled with RNA from recent migrants from robust HIV-1 populations in the blood circulation, and because (ii) not all reservoirs necessarily give rise to HIV-1 RNA secreted into semen at all times. In support of the idea of the male genital tract as a potential reservoir, recent work has suggested that a substantial reservoir of HIV-1 persists during antiretroviral therapy in urethral macrophages in the male genital tract [24].

Most study participants in the current study, including the three individuals with this phenotype, received their primary care at the Ivan Toms clinic at which recruitment took place, and none had been offered ART before the samples were collected. All study participants were also asked to confirm that they were not on ART. Additionally, all three individuals with this phenotype were tested for antiretroviral drugs in two ways: (i) serum of each individual was tested for the ability to inhibit entry and subsequent gene expression of HIV-1 pseudotyped viruses expressing the envelope of murine leukaemia virus (MLV) [25]; (ii) blood plasma was tested for the presence of efavirenz and lopinavir by liquid chromatography tandem mass spectrometry (LC MS/MS, qualitative test with limit of detection 0.02 μg/ml for both drugs). Efavirenz and boosted lopinavir were the standard backbones for first and second line ART, respectively, for adult men receiving ART in the public sector in South Africa at the time of recruitment. The limit of detection of the antiretroviral assays is considerably lower than the minimum trough concentration recommended for efficacy (1 μg/ml for both drugs [26]).

Antiretroviral drugs were not detected by either method. The inability to inhibit MLV pseudotyped HIV-1 suggests that no antiretroviral drug was present at functional concentrations. The LC MS/MS result indicates that it is highly unlikely that efavirenz in normally prescribed doses was taken in the 7 days prior to sample collection or lopinavir in the 24 h prior to sampling. The time window is wider for efavirenz because of its longer half-life [27].

The presence of HIV-1 RNA in seminal plasma of individuals whilst on ART has been better studied than in ART naive individuals. HIV-1 is detectable in seminal plasma, at least transiently, in approximately 5-10% of men living with HIV-1 who were on ART with undetectable blood viral loads [1–5]. This presence of HIV-1 RNA in semen of ART-treated males is usually not associated with time since ART initiation, suggesting that this was not simply a slower decay of HIV-1 in semen compared to blood upon initiation of ART. It is not clear if this is related by mechanism to the HIV-1 shedding in semen of ART-naive individuals reported here.

Generally, in individuals on ART with suppressed blood viral loads (<200cp/ml), the presence of HIV-1 RNA in semen is not associated with a detectable transmission risk, even in MSM populations with high STI rates [7]. The results presented herein are nonetheless relevant to understanding the potential tissue reservoirs of HIV-1.

What controls HIV-1 RNA shedding into semen is unclear. Although limited penetration of ART into the male genital tract may play a role, the relationship appears complex and partial [11] and does not play a role in the ART naive individuals described in this report. Notably, in ART treated males, the presence of HIV-1 RNA in seminal plasma was associated with the presence of cytomegalovirus (CMV) DNA, in one study [3], but not another [5]. Separately, congenital CMV infection, presumably as a proxy measure for active CMV infection in the placenta, is a strong risk factor (odds ratio = 20) for mother to child transmission of HIV-1 in the face of widespread maternal ART [28]. These associations between active CMV infection and HIV-1 replication suggests that breakthrough HIV-1 replication may have a biological basis other than, or in addition to, poor penetration of ART drugs.

## Conclusion

That HIV-1 populations tend to be compartmentalized between the general circulation and the male genital tract is well established [29, 30]. Importantly, the results presented here suggest that independent HIV-1 replication in the male genital tract sometimes occurs, resulting in shedding of HIV-1 RNA into seminal plasma. Even highly effective immune responses resulting in extended periods with undetectable blood viral loads are not always sufficient to suppress this shedding. This replication could reflect that the male genital tract is the site of a persistent HIV-1 reservoir.

## Supplementary information

**Additional file 1: Figure S1.** Correlation between semen and blood viral load and presence of individuals with semen viral loads but low blood viral loads in previously published data. All graphs generated from table data in the reports except Olivier et al from supplemental figure 2B. Tachet et al analyzed 52 individuals incl 21 on ART & did not identify who was on ART. Red circles indicate individuals with low blood viral loads and detectable HIV-1 RNA in semen. None of the reports commented on VL data from these individuals. Note that x-axis scales do not always match and that the y-axis scale for Olivier et al is different from the other graphs. Graph from Olivier et al reprinted from J Infect Dis, 209, 1174–84 (2014), Olivier AJ et al, Distinct cytokine patterns in semen influence local HIV shedding and HIV target cell activation by permission of Oxford University Press.

## Data Availability

The data supporting the conclusions of this article are included within the article.

## Abbreviations

ART: Antiretroviral therapy
CMV: Cytomegalovirus
cp: Copies
DNA: Deoxyribonucleic acid
HIV-1: Human immunodeficiency virus-1
IQR: Interquartile range
LC MS/MS: Liquid chromatography tandem mass spectrometry
MLV: Murine leukaemia virus
MSM: Men who have sex with men
NSC: Nonsperm seminal cells
PBS: Phosphate buffered saline
RNA: Ribonucleic acid
STIs: Sexually transmitted infections
