# Supplementary figures and images for: Detectable HIV-1 in semen in individuals with very low blood viral loads
Source: Virol J. 2020 Mar 5;17:29. doi: 10.1186/s12985-020-01300-6 (PMC7059658; doi:10.1186/s12985-020-01300-6)

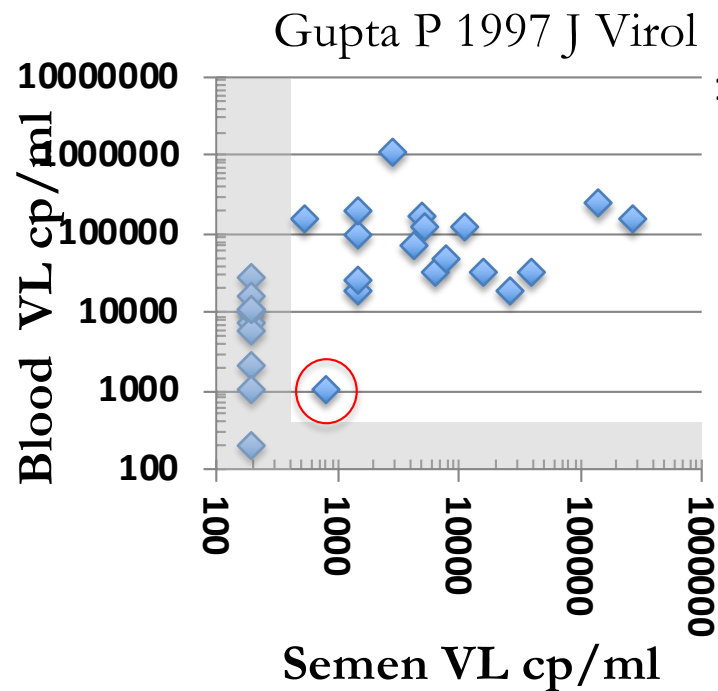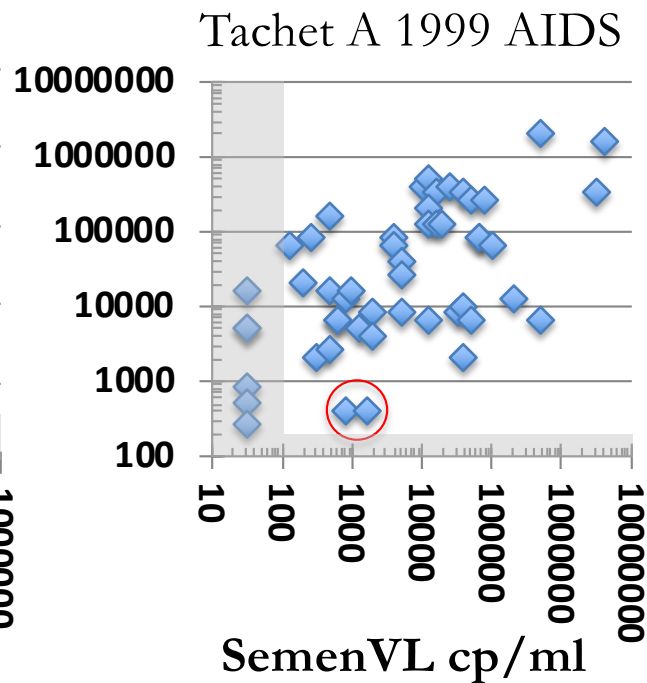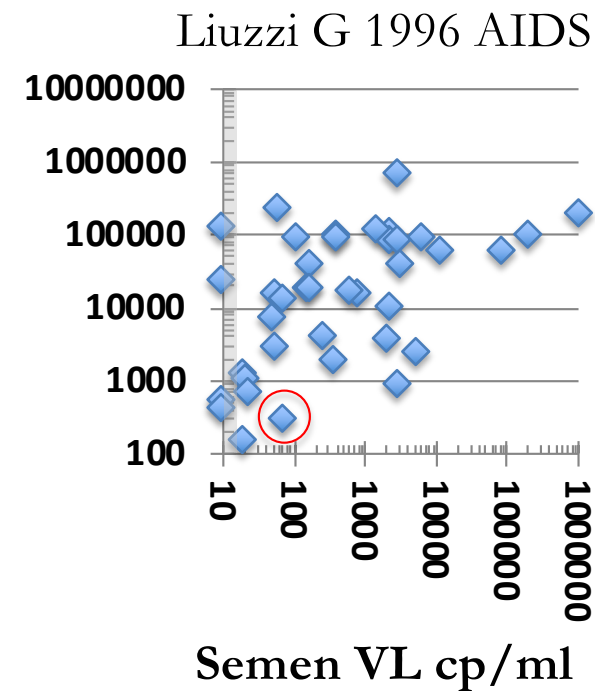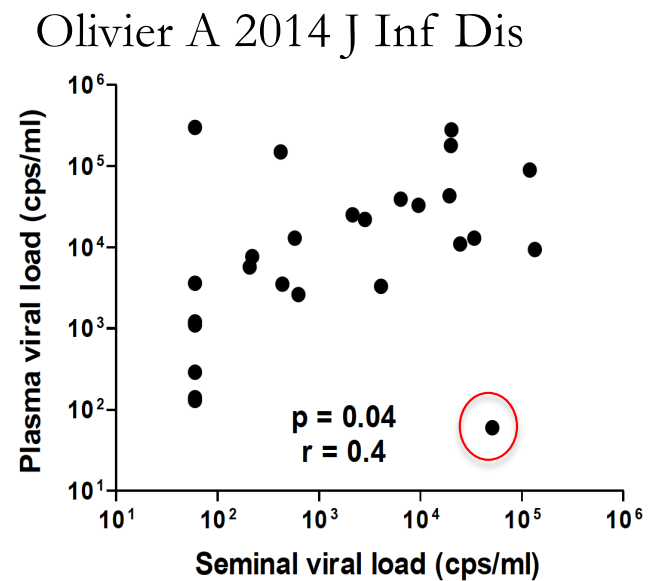

Supplement: Supplementary file 1 — Additional file 1: Figure S1. Correlation between semen and blood viral load and presence of individuals with semen viral loads but low blood viral loads in previously published data. All graphs generated from table data in the reports except Olivier et al from supplemental figure 2B. Tachet et al analyzed 52 individuals incl 21 on ART & did not identify who was on ART. Red circles indicate individuals with low blood viral loads and detectable HIV-1 RNA in semen. None of the reports commented on VL data from these individuals. Note that x-axis scales do not always match and that the y-axis scale for Olivier et al is different from the other graphs. Graph from Olivier et al reprinted from J Infect Dis, 209, 1174–84 (2014), Olivier AJ et al, Distinct cytokine patterns in semen influence local HIV shedding and HIV target cell activation by permission of Oxford University Press. [file 12985_2020_1300_MOESM1_ESM.pdf]
